# Palliative Sedation at Home in Patients with Advanced Cancer: A Retrospective Observational Study of Clinical Characteristics and Sedation-Related Outcomes

**DOI:** 10.3390/jcm15218494

**Published:** 2026-11-01

**Authors:** Salvador Martín Utrilla, Paula Oliver de Haro, Julián Mollá Marinas, Antonio Mancheño Álvaro, Enrique Jesús Sáez Álvarez

**Affiliations:** 1 Grupo de Investigación en Cuidados Paliativos (GRICPAL), Facultad de Medicina y Ciencias de la Salud, Universidad Católica de Valencia “San Vicente Mártir”, c/Espartero 7, 46007 Valencia, Spain; paulaodh@hotmail.com (P.O.d.H.); enriquejesus.saez@ucv.es (E.J.S.Á.); 2 Fundación Instituto Valenciano de Oncología, c/Beltrán Báguena 4, 46009 Valencia, Spain; amancheno@fivo.org

**Keywords:** palliative sedation, advanced cancer, hospital at home, refractory symptoms, palliative care

## Abstract

**Background/Objectives**: Palliative sedation may relieve refractory suffering in patients with advanced cancer receiving end-of-life care at home. We aimed to describe patient characteristics, palliative sedation use, refractory symptoms, and sedation-related outcomes in a specialized hospital-at-home unit. **Methods**: We conducted a retrospective observational study of patients with advanced cancer who died during 2022 while receiving care from the Instituto Valenciano de Oncología hospital-at-home unit, Spain. Admission required clinical and social assessment confirming the need for hospital-level home care, feasibility of care at home, and adequate caregiver support. Sociodemographic, clinical, and sedation-related data were obtained from medical records. Analyses were descriptive and exploratory; associations were unadjusted. **Results**: The cohort included 113 patients; 80 (70.8%) received palliative sedation. Median age was 76 years (IQR, 19), and 59 patients (52.2%) were men. An elastomeric infuser was used in 74 sedated patients (92.5%). Sedation duration had a median of 1 day (IQR, 2; range, 0–10). Symptom documentation was available for 56 sedated patients (70.0%); dyspnea was documented in 35 (62.5%), delirium in 19 (33.9%), and pain in 14 (25.0%). Symptoms could coexist. The median number of parenteral medications was 4 (IQR, 1). **Conclusions**: Palliative sedation was frequently used in this selected specialized home-care cohort and generally lasted a short period. The prevalence should not be generalized to other settings. Incomplete documentation and the retrospective, single-centre design limit interpretation. Prospective multi-centre studies are needed.

## 1. Introduction

Hospital at Home (HaH) is a healthcare model that provides hospital-level medical and nursing care at home, offering services comparable to conventional hospitalization [1]. In Spain, HaH has significantly expanded since the establishment of the first unit in Madrid in 1981 [2], with over 130 units currently operating nationwide [3]. However, the distribution and scope of services vary across autonomous communities. In the Valencian Community, there are 27 HaH units, including the Instituto Valenciano de Oncología (IVO), where all patients present oncological pathologies [4]. In many HaH units, advanced palliative care is crucial, enabling patients with advanced diseases to remain at home with optimal symptom management. While this type of care is central at IVO, other units manage a wider range of pathologies. A 2023 study in the Basque Country reported that 26% of HaH patients required palliative care, with some units reporting up to 39%, reflecting considerable variability [1].

The primary goal of Palliative Care in HaH is to provide effective symptom control, allowing patients to spend their final days without suffering in a dignified environment while avoiding unnecessary hospitalizations [5]. Despite advancements in this care model, home-based end-of-life care remains a challenge in many healthcare systems, even though it is the preferred option for many patients and families. Thus, specialized professionals capable of identifying and managing refractory symptoms at home while considering the home environment are needed [6]. According to the World Health Organization (WHO), approximately 40 million people could benefit from palliative care due to the increasing incidence of cancer. Proper follow-up by specialized professionals ensures symptom control. However, in some cases, symptoms remain unmanageable despite available treatments, leading to “refractory symptoms” [7].

Patients in the last days of life often experience refractory symptoms, with delirium or agitation, pain, dyspnea, death rattle, and psychological distress being the most common [7,8,9,10]. In such cases, the supervised administration of medications to reduce patient consciousness and alleviate existential and physical suffering is termed palliative sedation [7,11,12]. The most frequently used medications for palliative sedation include midazolam and levomepromazine, with haloperidol, morphine, scopolamine, and hyoscine also frequently administered. On average, 4.3 drugs are used for symptom control in the final phase of life [7,9].

Several studies have analysed the use of palliative sedation at HaH. A study on home-based oncological patients reported an average duration of care of 63.9 days, with 66% of patients dying at home. Among these patients, 12% required palliative sedation due to refractory symptoms, primarily delirium and dyspnea. The most common cancer types are gastrointestinal, pulmonary, and genitourinary. The mean time from sedation initiation to death was 2.6 days [9]. Another study [10] reported that 56.34% of patients required palliative sedation, with continuous infusion being used in 94% of patients. Dyspnea (22%) and delirium (18%) were the most common symptoms, with midazolam being the most used drug. A recent study (2024) [7] revealed that 16.7% of patients required palliative sedation, with a mean duration of two days until death, and 98% required continuous sedation. The most frequent symptoms were delirium (36.1%), pain (31.9%), and dyspnea (25%).

Few studies have compared palliative sedation in hospital versus home settings. A study in Italy suggested that, while symptom prevalence varies between care settings, appropriate management can be effectively implemented in both. This highlights the importance of adapting palliative care protocols to home settings to ensure optimal patient care without hospitalization [13].

Early palliative care for patients with advanced cancer should not only include timely symptom assessment and psychosocial support, but also anticipatory planning for the later stages of illness. This planning may involve discussing the preferred place of care, evaluating the home environment and caregiver capacity, identifying the expected need for hospital-level home care, and preparing patients and families for the possible emergence of refractory symptoms and the potential need for palliative sedation. In this context, understanding the clinical and organizational characteristics of patients who receive end-of-life care at home may help palliative-care teams identify care needs earlier and establish appropriate referral and support pathways.

In recent years, an increasing number of advanced cancer patients have preferred to remain at home during their final stages, receiving home-based palliative care. HaH has proven to be an effective alternative, offering better symptom control, avoiding unnecessary transfers, and ensuring appropriate support within the family environment. Despite the growing role of HaH in managing these patients, studies focusing on the characteristics of the home-based patient population and the interventions provided in the terminal phase of life are lacking.

Within the framework of the Spanish Society of Hospital at Home, a multi-centre study on end-of-life care in this care setting was proposed. As a participating centre, we analysed data from our hospital, the Instituto Valenciano de Oncología. The aim of this study was to describe the clinical and sociodemographic characteristics of patients with advanced cancer receiving home-based hospital care, to characterize the use of palliative sedation and the refractory symptoms leading to its initiation, and to explore unadjusted associations between patient characteristics and sedation-related outcomes. By collecting data, we seek to optimize care quality and facilitate decision-making in home-based end-of-life care, allowing for protocol adjustments and ensuring personalized care tailored to each patient’s needs.

## 2. Methodology

A retrospective observational study was conducted, including patients who died between 1 January and 31 December 2022, under the care of the HaH Unit of the Instituto Valenciano de Oncología (IVO). Patients without a documented “palliative care” status in their clinical history or those transferred to a hospital for end-of-life care were excluded. Additionally, patients lacking medical records for data collection were also excluded. The study was conducted in a specialized hospital-at-home unit serving patients with advanced cancer. Admission was not open to all patients receiving palliative care. Patients were accepted after an individualized clinical and social assessment confirming the need for hospital-level home care, the feasibility of managing the patient at home, and the availability of adequate family or caregiver support. Before admission, the patient’s suitability was assessed by at least two physicians. The assessment considered the patient’s clinical condition, symptom burden, expected care needs, home environment, caregiver availability, and the feasibility of providing end-of-life care at home. Accordingly, the study population represents a selected group of patients admitted to a specialized home-care pathway and should not be considered representative of the broader population of patients receiving palliative care at home.

Sociodemographic and clinical data were collected from patient records and routine hospital activity logs. A nonprobabilistic convenience sampling method was used, and all eligible patients were selected on the basis of predefined inclusion and exclusion criteria. Data collection adhered to current legislation on personal data protection and was subsequently transferred to a computerized database.

Ethical and legal requirements were met, following Good Clinical Practice principles (*Real Decreto* 561/1993, Ministry of Health and Consumer Affairs) and the Helsinki Declaration. Informed consent was not needed, as this was a retrospective study of deceased patients, ensuring full anonymization of personal data. Ethical approval was granted by the promoting hospital’s Research Ethics Committee in December 2023.

Basic sociodemographic variables, including sex, age, marital status, usual residence, and living arrangements of the patients, were recorded. Additionally, healthcare-related variables were collected, such as the identification of the primary caregiver, the inclusion of an advanced care plan, the need for palliative care in the referral report, the length of stay in the unit, and the need for palliative sedation at the end of life. Length of stay and number of palliative-care team visits were reported descriptively. Because the original analysis did not include a sufficiently documented assessment of parametric assumptions for these variables, no inferential comparison between sedation groups was retained.

Functional status was assessed via the Eastern Cooperative Oncology Group (ECOG) Performance Status. The Barthel Index was used to evaluate the ability to perform basic activities of daily living (eating, bathing, dressing, etc.), categorizing patients as independent (BI: 90–100), moderate dependence (BI: 60–89), or severe dependence (BI < 60). Additionally, the Global Deterioration Scale (GDS) was employed to assess cognitive function, classifying patients according to their degree of cognitive decline as mild (GDS < 4), moderate (GDS: 4–5), or severe (GDS: 6–7). Other recorded variables included the type of neoplasm, the presence of geriatric syndromes (constipation, delirium, malnutrition, pressure ulcers, and urinary incontinence), the presence of polypharmacy (use of five or more daily medications), opioid use upon admission and its morphine-equivalent dose, and the presence of multimorbidity.

Palliative sedation was considered in patients with advanced cancer in the terminal phase of illness who experienced severe suffering due to one or more symptoms that were refractory to conventional treatment. For the purpose of this study, a refractory symptom was defined as a symptom that caused significant suffering and could not be adequately relieved despite appropriate treatment, or for which further treatment was considered unlikely to provide adequate relief within the patient’s expected timeframe. The decision to initiate sedation was individualized and based on the clinical assessment of the physician responsible for the patient within the home-hospitalization/palliative-care team. The assessment included the patient’s clinical condition, prognosis, symptom severity, previous therapeutic interventions, goals of care, and available advance-care planning information. Nursing staff participated in clinical assessment, medication administration, and follow-up. When clinically feasible, the patient and/or family caregivers were informed about the indication for sedation, its intended effect on consciousness, and the expected course. The decision was made within a shared decision-making process involving the responsible physician, the patient whenever clinically able to participate, and the family or designated caregivers when appropriate. The patient’s previously expressed preferences and advance-care planning documentation were considered when available.

Sedation was initiated with the aim of relieving refractory suffering, not of hastening death. Medication choice, administration device, dose, and subsequent adjustments were determined according to the patient’s symptoms and clinical response. Follow-up was based on repeated clinical assessment documented in the medical record, including symptom evolution, level of consciousness, respiratory status, medication administration, and the need for dose adjustment or rescue medication. Because this was a retrospective study, validated sedation-depth and symptom-control scales were not systematically available for all patients. In the available clinical documentation, monitoring focused on the patient’s observable clinical response, persistence or relief of the refractory symptom, level of consciousness, respiratory pattern, medication administration, and the need for dose changes or rescue medication. However, the retrospective design did not allow us to determine whether these domains were assessed using a uniform protocol in every case. Validated sedation-depth scores and standardized symptom-control measures were not consistently recorded.

### 2.1. Variables Related to Palliative Sedation (PS)

Variables related to palliative sedation, including medication administration devices (elastomeric infusers or electric systems), were also recorded. Refractory symptoms leading to sedation, such as delirium, dyspnea, pain, active bleeding, vital emotional suffering, or other symptoms, were documented. Additionally, the number of required parenteral medications and the duration of palliative sedation until death were recorded.

### 2.2. Statistical Analysis

For the descriptive analysis, frequencies and percentages were calculated for qualitative variables, whereas means and standard deviations were computed for quantitative variables following a normal distribution. When variables did not follow a normal distribution (Kolmogorov–Smirnov test or Shapiro–Wilk test for samples ≤ 50), the median and interquartile range (IQR) were reported. These analyses were performed for the entire sample or differentiated by study groups, as appropriate.

For bivariate analysis, appropriate statistical tests were applied to evaluate the relationships between two variables. If both variables followed a normal distribution and were numerical, Pearson correlation coefficients and/or linear regression were used. If one variable was quantitative and the other was qualitative, Student’s *t* test was applied when the qualitative variable had two levels, and ANOVA was used when it had three or more levels.

If the variables did not follow a normal distribution, when both were numerical, Spearman’s correlation was used. When one was quantitative and the other was qualitative, the Mann–Whitney U test was applied for two-level qualitative variables, and the Kruskal–Wallis test was used for those with three or more levels. If both were qualitative, no normality check was needed, and either the χ^2^ test (for r × s tables) or Fisher’s exact test (for 2 × 2 tables) was used.

No multivariable logistic regression model was fitted for the outcome of receipt of palliative sedation because of the limited sample size, the imbalance between groups, and the exploratory nature of the study. Therefore, all reported associations should be interpreted as unadjusted and exploratory. Sedation duration was summarized descriptively using the median, interquartile range, minimum, and maximum because the variable was discrete and non-normally distributed. Exploratory comparisons were performed using non-parametric methods where appropriate. Because of the limited sample size and the distributional characteristics of sedation duration, no regression model was used to identify independent factors associated with the duration of sedation. Sedation duration was summarized descriptively using the median, interquartile range, minimum, and maximum because the variable was discrete and non-normally distributed. Given the limited sample size and the distributional characteristics of the outcome, no regression or correlation analysis was used to identify independent factors associated with sedation duration. These analyses were considered secondary and hypothesis-generating.

## 3. Results

### 3.1. Demographic and Healthcare Characteristics of the Population

The study sample consisted of 113 patients (Table 1), of whom 59 were men (52.2%) and 54 were women (47.8%). The median age was 76 years (IQR 19), with a range between 43 and 95 years. The median age for men was 77 years (IQR 14), whereas for women, it was 75 years (IQR 23). No significant differences in age were found between men and women (Mann–Whitney U test, *p* = 0.277).

With respect to marital status, data were available for 79 patients, of whom 46 (40.7%) were married, 24 (21.2%) were widowed, and 9 (8%) fell into other categories. Concerning usual residence, data were unavailable for 15 patients. Among the remaining patients, 87 (77%) lived in their own homes, 11 (9.7%) lived with other relatives, and none resided in a long-term care facility. In terms of living arrangements, the variables were recategorized into four groups: living with direct family members (spouses, partners, or children), living with a family member and a caregiver, being cared for by an external caregiver, and having no designated caregiver. Among the 113 patients, 74 (66.4%) lived with family members, 7 (6.2%) lived with a family member and a caregiver, 7 (6.2%) were under the care of an external caregiver, and 24 (21.2%) had no specific caregiver. Among 82 patients (72.6%), a family member was actively involved in their care, whereas 14 (12.4%) relied on an external caregiver.

No significant differences by sex were found in relation to age (Mann–Whitney U test, *p* = 0.277), usual residence (χ^2^ = 0.574, *p* = 0.750), living arrangements (χ^2^ = 4.132, *p* = 0.127), or marital status (χ^2^ = 0.356, *p* = 0.069), suggesting a homogeneous distribution of patients regardless of sex.

At the time of admission to the unit, 94 patients (83.2%) had a referral report that included the need for palliative care. Additionally, only 15 patients (13.3%) had an advanced care plan documented in their medical history. The mean length of stay in the unit was 47.5 days (SD = ±55.8).

The mean length of stay in the unit was 47.5 days (SD = 55.8). Descriptively, the mean length of stay was 37.3 days (SD = 44.5) among patients who did not receive palliative sedation and 51.7 days (SD = 60.9) among those who did. Because no inferential comparison was retained, these descriptive differences should not be interpreted as evidence of a statistically significant difference between groups.

The mean number of visits by the palliative-care team was 20.6 (SD = 27.4). Descriptively, the mean number of visits was 14.9 (SD = 14.5) among patients who did not receive palliative sedation and 23.1 (SD = 31.2) among those who did. No inferential comparison between groups was retained because the assumptions underlying parametric testing had not been sufficiently documented.

The clinical characteristics at admission, such as primary tumour site, Barthel Index, ECOG-PS, GDS, multimorbidity conditions, geriatric syndromes, polypharmacy and use of opioids, are shown in Table 2.

### 3.2. Characteristics of Palliative Sedation

Palliative sedation was required in 80 patients (70.8%), whereas 33 (29.2%) did not require sedation. The most used sedation device was the elastomeric infuser, which was employed in 74 patients (92.5%). The remaining 6 patients (7.5%) received sedation through an electric system.

Sedation duration did not follow a normal distribution (K-S = 0.278, *p* < 0.005), with a median of 1 day (IQR, 2; range, 0–10). The most common durations were as follows: 1 day in 37 patients (46.3%), 2 days in 13 patients (16.3%), and same-day death after sedation in 10 patients (12.5%). No significant relationship was observed between sedation duration and sex (Mann–Whitney U test, *p* = 0.746).

Among the 80 patients who received palliative sedation, documentation of the refractory symptom(s) leading to sedation was available for 56 patients (70.0%) and was missing for 24 patients (30.0%). Dyspnea was documented in 35 patients (62.5% of those with available information), pain in 14 patients (25.0%), delirium in 19 patients (33.9%), active bleeding in 2 patients (3.6%), and vital emotional suffering in 5 patients (8.9%). Symptoms were not mutually exclusive and could therefore overlap. These data are detailed in Table 3.

The number of medications used for sedation did not follow a normal distribution (K-S = 0.352, *p* < 0.0001), with a median of 4 drugs, a minimum of 1, a maximum of 7, and an IQR of 1. A linear relationship was identified between the number of drugs used and sedation duration (Spearman’s Rho r = −0.253, *p* = 0.023), indicating that a greater number of medications was associated with shorter survival after sedation initiation.

Several clinical variables, including multimorbidity, geriatric syndromes, medication burden, Barthel Index, ECOG Performance Status, and cognitive status, were examined in relation to receipt of palliative sedation. However, no statistically significant unadjusted association was consistently demonstrated between these variables and the need for sedation.

Among the 113 patients, 87 (77%) had two or more chronic diseases, whereas 26 (23%) had no multimorbidity. Only one patient did not have an oncological disease (Category G), whereas 112 patients (99.1%) were oncology patients without disease-modifying treatment. The most frequent category was G, followed by F (20 patients, 17.7%) and B (15 patients). Multimorbidity was not associated with the need for sedation (χ^2^, *p* = 0.474) or with sedation duration (Mann–Whitney U test, *p* = 0.881).

Geriatric syndromes were present in 38 patients (33.6%). This composite variable was defined as the presence of at least one of the following conditions recorded at admission: constipation, delirium, malnutrition, pressure ulcers, or urinary incontinence/urinary catheter use. The individual frequencies are defined in Table 2. Because patients could present with more than one condition, the component frequencies were not expected to sum to the number of patients with at least one geriatric syndrome. No statistically significant unadjusted association was observed between the presence of geriatric syndromes and receipt of palliative sedation (*p* = 0.710) or sedation duration (*p* = 0.532).

Polypharmacy, defined as the use of five or more medications at admission, was present in 61.1% of patients (69). The median number of medications was 6 (IQR 5; range 0–19). No statistically significant association was observed between polypharmacy and receipt of palliative sedation (*p* = 0.376) or disease duration (r = −0.119, *p* = 0.292).

Upon admission to home-based care, 31.9% of patients (36 out of 113) were already using opioids. The most administered daily morphine-equivalent dose (MED) was 10 mg, followed by 5 mg and 60 mg. No associations were found between sedation duration and the opioid dose (Mann–Whitney U test, *p* = 0.088) or between the opioid dose and the need for sedation (χ^2^, *p* = 0.264). Sedation duration was therefore reported descriptively, and no independent factor associated with its duration was identified.

## 4. Discussion

There is no doubt that home care is an area of special interest in the treatment of individuals, especially in the final stages of their lives. Several factors contribute to the need to address this topic from multiple perspectives, areas, and strategies. This article aims to make a significant contribution to this ongoing reality, to the urgent need for knowledge that can no longer wait, because it is part of the daily lives of an increasing number of people. The scientific literature indicates that the home is the preferred place for many patients to die [14,15,16,17,18]. Although home may be an important setting for end-of-life care, our study cannot estimate the proportion of patients who preferred to die at home. A preferred place of death was documented in only 24 of the 113 patients (21.2%), and the distribution of the documented preferences was not consistently available. Therefore, these data should not be interpreted as representative of the entire cohort, which greatly increases the need to understand the reality of home care. However, to date, we do not have much published experience regarding the process and outcomes of home palliative sedation care.

The prevalence of palliative sedation in this cohort was high (70.8%). This figure should be interpreted in the context of the study setting and not extrapolated to all patients receiving home-based palliative care. Patients were admitted to a specialized hospital-at-home unit only after clinical and social assessment, including evaluation by at least two physicians, confirmation of advanced illness and hospital-level care needs, and assessment of the feasibility of providing care at home with adequate caregiver support. This admission pathway may have enriched the cohort with patients in advanced or terminal stages who were more likely to require complex symptom management and palliative sedation.

The prevalence may also reflect institutional factors, including the oncology-focused case mix, referral patterns, local clinical protocols, staff expertise, access to home visits, and the availability of caregiver support. Conversely, patients transferred to hospital before death were excluded, which may have further influenced the observed proportion. Therefore, the 70.8% prevalence should be regarded as a setting-specific descriptive estimate rather than a general population estimate.

In the analysis presented here, with a range between zero and ten days, the median duration of palliative sedation was one day, with three-quarters of the patients dying during this period, a finding very similar to those reported by other authors with samples of oncology patients treated at home [19] or in the general population [20].

Length of stay and the number of palliative-care team visits were reported descriptively. These variables provide contextual information about the organization of home-based care but do not allow conclusions regarding the adequacy, quality, or effectiveness of palliative sedation. Because standardized measures of symptom control, sedation depth, breakthrough symptoms, rescue medication, and patient comfort were not consistently available, the quality of sedation could not be evaluated reliably.

Sedation duration was described using non-parametric summary measures because of its discrete and non-normal distribution. We did not attempt to identify independent factors associated with sedation duration. Although functional status may be clinically relevant, the available data and sample size did not support a reliable multivariable or regression-based assessment. Any potential relationship between functional status and sedation duration should therefore be evaluated in larger prospective studies using standardized measurements and prespecified models.

Among patients with available symptom documentation, dyspnea was the most frequently recorded refractory symptom associated with the initiation of palliative sedation, followed by delirium. Because symptom information was missing for 30% of sedated patients, these findings should be interpreted as descriptive and exploratory. These data are partially similar to those offered by Calvo et al. [19] or Mercadante et al. [21], with similar samples, who primarily highlight delirium.

In this selected cohort of patients with advanced cancer receiving specialized Hospital-at-Home care, palliative sedation was frequently used. The median age was 76 years, polypharmacy was present in 61.1% of patients, and geriatric syndromes were documented in 33.6%. Among patients with available symptom documentation, dyspnea was the most frequently recorded refractory symptom. The median duration of palliative sedation was 2 days (IQR, 2; range, 0–10). These findings are descriptive and setting-specific and should not be interpreted as causal or generalizable estimates. This is consistent with the review by García et al. [22] in 2023, which was based on a series of 23 studies that ranked the frequency of refractory symptoms in descending order: pain, dyspnea, and delirium. Because symptom documentation was missing for 24 of the 80 sedated patients, this finding should be interpreted cautiously.

Several limitations should be considered. First, this was a retrospective, single-centre study with a relatively small sample and a nonprobabilistic sample of patients receiving specialized home-based care. Second, the marked imbalance between patients who received palliative sedation and those who did not, together with missing data for some clinical variables, limited the feasibility and reliability of a multivariable model. Third, the statistical analyses were mainly descriptive and bivariate; therefore, the reported associations were unadjusted and cannot be interpreted as independent determinants or causal relationships. Fourth, the medical records did not consistently contain standardized assessments of sedation depth, symptom intensity, breakthrough symptoms, rescue medication, dose escalation, or episodes of distress during sedation. Consequently, the present study cannot provide a robust assessment of sedation efficacy, equivalence of sedation depth, or quality of symptom control. Finally, the findings may not be generalizable to other home-hospitalization units or healthcare systems with different referral patterns and sedation protocols. The retrospective design resulted in incomplete documentation of patients’ preferences regarding their preferred place of death. This information was available for only 24 of 113 patients (21.2%). Consequently, the study cannot estimate the prevalence of a preference for dying at home, and the available records may not be representative of the entire cohort. This limitation also prevents a robust assessment of the concordance between patients’ preferences and the place in which end-of-life care was ultimately provided.

It is important to mention the possibilities for improvement in this study. One of them is that it would have been desirable to include in the analysis the study of patients’ “awakenings” with discomfort during sedation, incidental pain episodes, the need for rescue interventions, or the need for dose increases owing to lack of symptomatic control, with the goal of having a more complete map of the actual quality of sedation in the evaluated patient. Although functional status may be clinically relevant to the course of end-of-life care, the present study was not designed or powered to determine whether the Barthel Index was associated with sedation duration. This question should be addressed in larger prospective studies using standardized measurements and prespecified statistical models.

The findings are also relevant to the organization of early palliative care. Although the present study did not evaluate an early palliative-care intervention, it highlights several issues that should ideally be addressed before the terminal phase of illness. These include anticipating the potential need for hospital-at-home care, assessing caregiver availability and the home environment, discussing the preferred place of care, reviewing treatment goals, and preparing patients and families for the possible development of refractory symptoms and the use of palliative sedation when clinically indicated.

From this perspective, home-based end-of-life care should not be regarded as an isolated intervention initiated only during the final days of life. It may be more appropriately understood as part of a continuum of palliative care that requires earlier communication, advance-care planning, coordination between oncology and palliative-care teams, and timely referral to specialized home-care services. The high proportion of patients receiving sedation in this selected cohort further emphasizes the importance of anticipating complex symptom-management needs rather than considering them only when the patient is already in the terminal phase. The available data describe the organization of home-based care but do not permit an assessment of the quality, adequacy, or effectiveness of palliative sedation.

Finally, although the scientific literature presents some ambiguity regarding the use of the term palliative sedation [13,23,24], this study follows the definition of the European Association for Palliative Care, which is the use of sedative medications in terminal patients to alleviate intolerable suffering from refractory symptoms by reducing the patient’s level of consciousness [12].

## Figures and Tables

**Table 1:** Demographic and healthcare characteristics of the total population (N = 113).

Variables	Total (N = 113)	No PS (N = 33)	PS (N = 80)
Age			
Median (IQR)	76 (19)		
Sex, n (%)			
Male	59 (52.2)	19	40
Female	54 (47.8)	14	40
Marital status, n (%)			
Married	46 (40.7)	12 (36.3)	34 (42.5)
Widowed	24 (21.2)	9 (27.2)	15 (18.7)
Others/Not available	43 (38.1)	12 (36.3)	31 (38.7)
Residence, n (%)			
Own home	87 (77)	28 (84.8)	59 (73.7)
Other family members’ home	11 (9.7)	2 (6)	9 (11.2)
Not available	15 (13.3)	3 (9)	12 (15)
Identification of the primary caregiver in medical history, n (%)	88 (77.9)	26 (78.7)	62 (77.5)
Identification of palliative care needs in the referral report, n (%)	94 (83.2)	26 (78.7)	68 (85)
Inclusion of an advanced care plan in medical history, n (%)	15 (13.3)	3 (9)	12 (15)
Record in the medical history about the patient’s preferred place of death, n (%)	24 (21.2)	7 (21.2)	17 (21.2)
Length of stay, mean (SD), days	47.5 (±55.8)	37.3 (±44.5)	51.7 (±60.9)
Number of team visits, mean (SD)	20.6 (±27.4)	14.9 (±14.5)	23.1 (±31.2)
Palliative sedation (PS), n (%)	80 (70.8)		

N: sample size; PS: palliative sedation; SD: standard deviation. Values are presented descriptively. No inferential comparison between groups was retained for these variables because the assumptions for parametric testing were not sufficiently documented.

**Table 2:** Clinical characteristics at admission of the total population (N = 113).

Variables	Total (N = 113)	No PS (N = 33)	PS (N = 80)
Primary tumour site, n (%)			
Digestive	35 (31)	11 (33.3)	24 (30)
Breast	15 (13.3)	4 (12.1)	11 (13.7)
Gynaecologic	9 (8)	1 (3)	8 (10)
Lung	11 (9.7)	4 (12.1)	7 (8.7)
Urologic	27 (23.9)	9 (27.7)	18 (22.5)
Others	16 (14.2)	4 (12.1)	12 (15)
Barthel index, n (%)			
Independent	8 (7.1)	1 (3)	7 (8.7)
Moderate dependence	41 (36.3)	13 (39.3)	28 (35)
Complete dependence	64 (56.6)	19 (57.58)	45 (56.2)
ECOG Performance Status, n (%)			
0	3 (2.7)	0	3 (3.7)
1	16 (14.2)	3 (9)	13 (16.2)
2	24 (21.2)	9 (27.2)	15 (18.7)
3	37 (32.7)	10 (30.3)	27 (33.7)
4	33 (29.2)	11 (33.3)	22 (27.5)
Global Deterioration Scale (GDS) Index, n (%)			
Mild Cognitive Deterioration	87 (77)	27 (81.8)	60 (75)
Moderate Cognitive Deterioration	19 (16.8)	3 (9)	16 (20)
Severe Cognitive Deterioration	7 (6.2)	3 (9)	4 (5)
Multimorbidity condition, n (%)	87 (77)	24 (72.7)	63 (78.7)
Geriatric syndromes, n (%)	38 (33.6)	10	28
Incontinence/Urinary catheter	35 (24.8)	9	26
Constipation	30 (21.3)	11	19
Malnutrition	16 (11.3)	4	12
Pressure ulcers	11 (7.8)	3	8
Polypharmacy, n (%)	69 (61.1)	21 (63.6)	48 (60)
Use of opioids, n (%)	36 (31.9)	8 (24.2)	28 (35)
Mean equivalent dose of morphine in mg (SD)	57.1 (±69.2)	43.1 (±25.3)	61.3 (±77.6)

N: sample size; PS: Palliative sedation; SD: Standard Deviation; ECOG: Eastern Cooperative Oncology Group. Percentages were calculated using the total number of patients with available data for each variable. Geriatric syndromes were coded as present when at least one prespecified syndrome was documented. Individual geriatric syndromes were not mutually exclusive; therefore, their frequencies may overlap and may not sum to the composite variable.

**Table 3:** Palliative sedation characteristics (N = 80).

Variables	Value n (%)	Mean (SD)	Median (IQR)
Administration device			
Elastomeric infuser	74 (92.5)		
Electric system	6 (7.5)		
Refractory symptoms motivating sedation			
Delirium	19 (33.9)		
Dyspnea	35 (62.5)		
Pain	14 (25)		
Active bleeding	2 (3.6)		
Vital emotional suffering	5 (8.9)		
Others	38 (67.9)		
Missing symptom documentation	24		
Number of refractory symptoms			
1	56 (70)		
2	17 (21.3)		
3	6 (7.5)		
4	1 (1.3)		
Number of parenteral drugs required		3.4 (1)	
Days of duration until death			1 (2)

Symptom information was available for 56 of the 80 sedated patients (70.0%). Percentages are calculated using this denominator. Symptoms were not mutually exclusive; therefore, percentages do not necessarily sum to 100%. The 24 patients without documented symptom information are reported separately as missing.

## Data Availability

The data presented in this study are available on request from the corresponding author, with permission of Fundación Instituto Valenciano de Oncología, but restrictions apply to the availability of these data, which were used under license for the current study, and so are not publicly available.
